# Reaching and targeting more effectively: the application of market segmentation to improve HIV prevention programmes

**DOI:** 10.1002/jia2.25318

**Published:** 2019-07-22

**Authors:** Anabel Gomez, Rebecca Loar, Andrea E Kramer, Geoffrey P Garnett

**Affiliations:** ^1^ AVAC New York NY USA; ^2^ Consultant Austin TX USA; ^3^ Consultant St. Petersburg FL USA; ^4^ Bill & Melinda Gates Foundation Seattle WA USA

**Keywords:** HIV prevention, segmentation, social marketing, demand creation, innovation, human‐centred design

Marketing techniques from the commercial sector, applied to the promotion of primary HIV prevention offer an opportunity to improve programme impact and deserve further exploration. One such technique, market segmentation, divides populations into groups and designs programmes that respond to groups’ distinct needs 1. Efficient and effective HIV prevention requires well targeted and well‐designed programmes responding to client characteristics and needs. Following best practices in commercial marketing would allow for more refined knowledge of those at risk and enable more tailored prevention interventions; specifically, the use of market segmentation that measures psychographic (psychological attributes such as values, attitudes and beliefs) and behavioural factors that might relate to product use and then describes segments of the population by their distinct needs, characteristics, or behaviours, facilitates the differentiation of products and/or marketing approaches by segment 2, 3. Market segmentation has recently been used in voluntary medical male circumcision (VMMC) programmes, providing a template worthy of consideration by other HIV prevention interventions 4, 5. This viewpoint argues that, well done, market segmentation should be an important component in developing and delivering HIV prevention interventions.

Successful market segmentation is a multi‐stage process, including: (1) qualitative work exploring the beliefs, attitudes, influences, habits and feelings of a population sample; (2) quantitatively surveying a representative sample of the population with questions derived from the qualitative analysis; (3) statistical analysis of survey data to generate non‐overlapping segments and narrative descriptions to distinguish how people in a given segment relate to potential products; (4) tailored programmes that appeal to each segment so they are persuaded to use the product; and (5) monitoring, rapid evaluation and correction when needed 2, 3, 6. In public health, market segmentation also considers who can benefit from a product or intervention, and the objective is not increased sales and profit, but the public good and a healthier population 1.

Segmentation is useful when it groups people according to characteristics that can be associated with marketing approaches, which in turn drive quantifiable outcomes 6. Segments should be identifiable, substantial, accessible and sustainable. In the context of HIV prevention, segments would need to be identifiable, discrete groups of those at risk of HIV, with attributes related to their beliefs, attitudes, influences, and habits related to HIV risk and HIV prevention. They should be substantial enough to make investments in products for HIV prevention and their promotion worthwhile. It should be easy, financially and emotionally, for those in the segment to access HIV prevention products and services. Finally, the risks of HIV and segment characteristics should be stable enough to allow for investments that would sustainably provide segment‐specific HIV prevention.

In our work on market segmentation, we have reviewed applications to public health and whilst some broad elements of segmentation, such as age and geography, have been employed, psychographics and behaviour have been less commonly used. This may be because psychographics, which measure client attitudes and interests, are harder, or more expensive to identify, and are seen as more subjective, less replicable and more prone to reporting bias than objective, demographic criteria. However, well‐applied psychographics provide a deeper understanding of the desires, needs and decision‐making considerations of a potential user of a product or service 3. Transferring methods and tools from the commercial to the public sector is likely also hampered by requirements for evidence by decision makers. In the commercial context, sales and profits provide rapid evidence; in public health, impact is typically observed in the medium‐ to long‐term.

Successful HIV prevention strategies to date share several features: political leadership, community engagement, attention to social norms and open communication 7. These successes have mainly been in key populations who have been engaged communities, highly motivated by HIV risk, or in more general populations responding to widespread HIV‐associated mortality 8. The current situation in southern and eastern Africa, where HIV is not the primary concern of young people may require additional approaches. Here, male and female condoms, VMMC and oral pre‐exposure prophylaxis (PrEP) are efficacious, but there are major barriers to uptake 9, 10. Designing social marketing strategies for these interventions, tailored for groups based on their values, attitudes and decision‐making creates the greatest likelihood that people will adopt them 5. The messages for behaviour change reducing numbers of risky sexual partnerships could also be tailored to specific segments, where the barriers to accessing and adopting prevention technologies are less, but the social contexts and norms may be challenging. There is experience of market segmentation in social marketing of condoms 11, but, to our knowledge, little has been documented and published in the peer‐reviewed literature. Table 1 describes the labels used to describe segments that have been used to categorize populations and the proportion of the population they represent in the few published studies from Malawi, Zimbabwe and Zambia 5, 12, 13. The segments provide information on the potential for success that interventions targeting particular segments might have, along with the scale of the benefits to be derived from successful campaigns. In addition, understanding the motivation of those within a category allows tailored messaging. For example, “scared rejectors” of circumcision would require interventions that address their fears, “friends‐driven hesitant” might respond to peer driven interventions. The studies of men's attitudes towards VMMC in Zambia and Zimbabwe revealed that the men responded best to information communicated individually rather than collectively, needed to overcome fear of pain, and needed to be prompted to attend the clinic 4, 13. These insights allowed Population Services International to develop specific VMMC campaigns through human‐centred design prototyping and testing 13. The approaches are now being applied across VMMC programmes and used in a cluster‐randomized trial to test the effectiveness of promotional materials informed by attitudes expressed by men engaged in the research. Defining the segments is a first step in the process of developing messages and interventions that need to be designed, trialled and delivered. Such intervention design should engage local communities to ensure acceptability and ownership.

**Table 1:** Examples of segments from HIV prevention studies

Perception of HIV risk and self‐efficacy (Malawi) 5		Condom attitude segments (Zimbabwe) 12		Attitude to VMMC among uncircumcised men (Zambia) 13		Attitude to VMMC amongst uncircumcised men (Zimbabwe) 13	
Responsive	35%	Playful	21%	Enthusiasts	21%	Socially supported believer	11%
Avoidance	8%	Caring	12%	Champions	6%	Self‐reliant believer	9%
Proactive	47%	Easy going	10%	Neophytes	19%	Knowledgeable hesitant	10%
Indifference	10%	Daring	17%	Scared rejectors	17%	Friends‐driven hesitant	19%
		Status^a^	19%	Embarrassed rejectors	16%	Scared rejector	17%
		Composed	21%	Highly resistant	21%	Indifferent resistant	27%
						Traditional believer	6%

a People concerned with their status in the community.

Building on work in VMMC, qualitative and quantitative surveys are underway amongst adolescent girls and young women (AGYW) in South Africa to explore attitudes to HIV prevention, generally and in terms of oral PrEP, and among young men and their attitudes around HIV testing 1. Experience shows that HIV prevention products are not automatically adopted by those at risk of HIV, but we believe that marketing best practices can increase the likelihood of success. Defining and describing segments is partly objective analysis and partly a creative narrative, the success of which can be judged by its utility in designing segment‐specific campaigns, in measuring the cost of those programmes, and ultimately in the uptake of prevention.

There is a need to study and document the identification of segments, the design of interventions to reach those segments, and the uptake of HIV prevention interventions within those segments.

## Competing interests

None.

## Authors’ contributions

A.G., R.L., A.E.K. and G.P.G. wrote this viewpoint collaboratively. All authors read and approved the final manuscript.
